# Supplementary material for: Cancerous phenotypes associated with hypoxia-inducible factors are not influenced by the volatile anesthetic isoflurane in renal cell carcinoma
Source: PLoS One. 2019 Apr 15;14(4):e0215072. doi: 10.1371/journal.pone.0215072 (PMC6464189; doi:10.1371/journal.pone.0215072)
Supplement: S1 File — File is deposited at https://doi.org/10.6084/m9.figshare.6571730. (DOCX) [file pone.0215072.s004.docx]

**Result of Data analysis of RNA-Seq**

File is deposited at https://doi.org/10.6084/m9.figshare.6571730.

FASTQ files for RCC4-EV cells (DRR100656) and RCC4-VHL cells (DRR100657) were obtained from the Sequence Read Archive (https://trace.ddbj.nig.ac.jp/dra/index_e.html). The quality of sequence data was evaluated by FastQC (http://www.bioinformatics.babraham.ac.uk/projects/fastqc/) after the trimming process by fastx_toolkit v 0.0.14 (http://hannonlab.cshl.edu/fastx_toolkit/). The human reference sequence file (hs37d5.fa) was downloaded from the 1000 genome ftp site (ftp://ftp.1000genomes.ebi.ac.uk/vol1/ftp/technical/reference/phase2_reference_assembly_sequence/), and the annotated general feature format (gff) file was downloaded from the Illumina iGenome ftp site (ftp://igenome:G3nom3s4u@ussd-ftp.illumina.com/Homo_sapiens/NCBI/build37.2/). The human genome index was constructed with bowtie-build in Bowtie v.2.2.9. The fastq files were aligned to the reference genomic sequence by TopHat v.2.1.1 with default parameters. Bowtie2 v2.2.9 and Samtools v.1.3.1 was used with the TopHat program47. Estimation of transcript abundance was calculated, and the count values were normalized to the upper quartile of the fragments per kilobase of transcript per million fragments mapped reads (FPKM) using Cufflinks (cuffdiff) v2.1.1. cuffdiff output (gene_exp. diff) was presentated (gene_exp.diff.txt).
